# Assessment of Serum CoQ10 Levels and other Antioxidant Markers in Breast Cancer

**DOI:** 10.31557/APJCP.2020.21.2.465

**Published:** 2020

**Authors:** Eman El-Attar, Amel Kamel, Ahmed Karmouty, Nadine Wehida, Rasha Nassra, Mohamed El Nemr, Noha Said Kandil

**Affiliations:** 1 *Department of Chemical Pathology, *; 2 *Department of Experimental and Clinical Surgery, *; 4 *Department of Medical Biochemistry, *; 5 *Department of Cancer Management and Research, Medical Research Institute, Alexandria University, *; 3 *Department of Pharmacology and Therapeutics, Pharos University in Alexandria, Alexandria, Egypt. *

**Keywords:** Breast cancer, CoQ10, Total antioxidant capacity, serum copper, serum zinc

## Abstract

**Background:**

The balance of the oxidative state in the body is fundamental for the maintenance of homeostasis. It has been implicated in the onset and progression of several diseases including breast cancer. The way in which the Reactive Oxygen Species (ROS) / antioxidants balance leads to or responds to disease is still controversial. In this study, TAC is used as a reference for the total antioxidant power of the body and Coenzyme Q10 (CoQ10) for its vital importance in cellular antioxidant action and being the only lipid soluble antioxidant synthesized endogenously. Copper and zinc were measured as trace elements reflecting the antioxidant micronutrient profile of the body.

**Methods:**

After approval of the ethical committee, 60 recently diagnosed non-intervened breast cancer patients were recruited from the Medical Research Institute hospital, Alexandria University along with 20 apparently healthy volunteers as control group. Full patient history was taken including breastfeeding history, parity, hormone replacement therapy use, body mass index, pathological examination, metastatic work up results, past medical history and drug use. CA 15-3 and laboratory investigations evaluating blood glucose, kidney and liver functions were performed. Q10 levels were measured by HPLC using a kit from Recipe®. TAC was assayed spectrophotometrically (Biodiagnostics®). Copper and Zinc levels were determined by inductively coupled plasma-optical emission spectrometry.

**Results:**

There was a statistically significant increase in the CoQ10, TAC and copper levels in the breast cancer group when compared to the control group. Zinc showed no statistically significant difference between the studied groups.

**Conclusion:**

Inspite of the fact that a high antioxidant level is usually considered as a favourable state, TAC, CoQ10 and copper levels showed significantly higher levels in the breast cancer group when compared to the control group. It is worth mentioning that the cancer group were all recently diagnosed, non-intervened and showed no signs of metastasis. It is still unclear whether the increased antioxidant levels offer a selective growth advantage to tumor cells over their surrounding normal cells or serve as a protective measure by the body in an attempt to correct the assault triggered by the ROS.

## Introduction

Breast cancer is the most common cancer in women. Its etiology and causative factors are complex and interlinked. Recognizable risk factors do not explain a substantial proportion of breast cancer cases, yet genetic studies have been found useful in identifying hereditary risk factors. It is still a challenge to identify individuals at risk who do not possess clear alarming risk factors.

Oxidative stress arises when there is an imbalance between reactive oxygen species (ROS) and antioxidants. It can make an impact on the different stages of cancer development (Jomova and Valko, 2011). It can alter the DNA sequence in the initiation step, increase cell division/ reduce apoptosis in the promotion stage and can also promote additional changes to the DNA in the progression stage (Reuter et al., 2010). 

Ubiquinone, a 1,4- benzoquinone, was recognized by Festenstein et al., (1955), then later named Coenzyme Q10 by Crane et al., (1957). It is also derived from the word ‘ubiquitous,’ signifying its highly abundant nature in the human cells. CoQ10 structure contains a 50 carbon isoprenoid side chain obtained both endogenously, through the mevalonate pathway and exogenously through the diet. It exists mainly in the reduced alcoholic form ‘ubiquinol- CoQ10H2’ and transforms readily to the oxidized ketonic ‘ubiquinone- CoQ10’ form ex vivo (Overvad et al., 1999). It is lipophilic in nature, being carried on lipoproteins in the circulation. CoQ10 plays a vital role in the electron transport chain. It enables the transfer of electrons and protons on oxygen, building up ATP as biochemical energy equivalents. It acts as a regenerating antioxidant transferring electrons from complexes I and II to complex III of the mitochondrial respiratory chain. It gets reduced from CoQ10 to CoQ10H2 by flavoenzymes, consequently donating the hydrogen atoms protecting cell contents from oxidation (Crane, 2001). Oxidative insult occurring in carcinogenesis has a debatable effect on the level of CoQ10 in the body. It is still unclear whether the fluctuations in the CoQ10 levels have a potential role in the etiology of breast cancer or is subsequent to the occurrence of breast cancer.

Total antioxidant capacity gives the advantage of quantifying all known and unknown antioxidants with the consideration of the synergistic interaction of these antioxidants, providing a fair evaluation of the antioxidant power in a provided sample (Ghiselli et al., 2000). It includes measuring enzymes such as superoxide dismutase, catalase and glutathione peroxidase; macromolecules such as albumin, ceruloplasmin and ferritin as well as an array of small molecules including ascorbic acid, α-tocopherol, β-carotene, reduced glutathione, uric acid and bilirubin (Bartosz, 2003). A reduced TAC level may occur as a result of the depletion of the antioxidant species in an attempt to correct the damage that has occurred to the tissues (Gupta et al., 2012). On the other hand, a raised antioxidant level may be observed as a protective measure to protect the body cells from the assault of the disease (Ray et al., 2000). 

Trace elements have a pivotal function in many biological processes. They are involved in the activation and inhibition of several reactions catalyzed by enzymes. It is suggested that the levels of trace elements in serum are linked to the incidence of breast cancer, yet the roles of different trace elements in the disease have not been adequately investigated (Gahlot et al., 2018). Copper generates ROS through the activation of different peroxides. The free radicals contribute to the mutations caused in the DNA resulting in their damage (Sîrbu et al., 2017). Hence, an increase in serum levels of copper could be a major factor in the onset of breast cancer. Zinc acts as an antioxidant. It has a protective function in the cell cycle against carcinogenesis. It also helps control gene transcription and plays an important role in the functions of many transcription factors and proteins that identify DNA sequences (Pisano et al., 2017). 

## Materials and Methods

*Study population and data collection*

After approval of the Ethical Committee, 80 patients were recruited from the Medical Research Institute, Alexandria University. They were divided into two groups, a group of 60 recently diagnosed non-intervened breast cancer patients and 20 apparently healthy volunteers as control group.Detailed history taking including general characteristics, thorough physical examination, anthropometric measurements to calculate their body mass index (BMI) and history of breastfeeding were recorded for all the participants. The TNM staging of the breast cancer patients were collected from the pathology reports after surgery and recorded as well as the metastatic work up results.

*Sample collection*

Five milliliters of blood were collected in serum tubes, left to coagulate for 30 minutes then centrifuged for 10 minutes at 3,000 rpm. The collected serum was divided into five aliquots, one for CoQ10 analysis, one for the total antioxidant analysis, the third for the CA 15-3 analysis, the fourth for the assay of zinc and copper and the fifth for the routine biochemical tests. The biochemical tests were analyzed on Olympus AU 400, clinical chemistry analyzer (Beckman Coulter Inc.) including determination of serum levels of random glucose, urea, creatinine, activities of aminotransferases, alkaline phosphatase. CA 15-3. CA15-3 was measured on IMMULITE 1000 immunoassay analyser (Siemens Healthcare and Diagnostics)., using a two-step sequential chemiluminescent immunometric assay.

*High Performance Liquid Chromatography*

**Figure 1:**
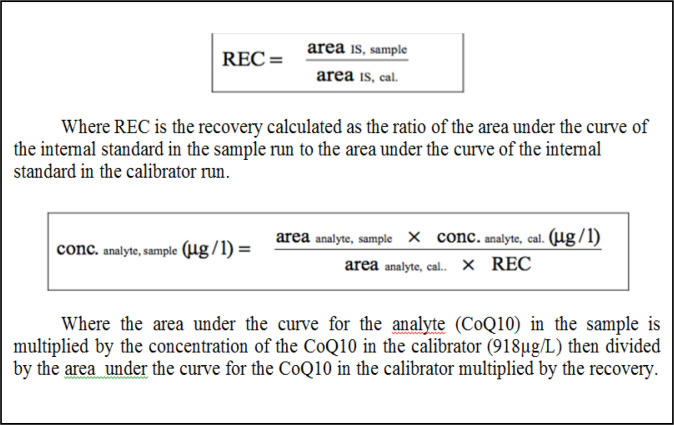
The Equations Used to Calculate then Recoveries and Concentrations of the Calibrator, Low Control and High Control

CoQ10 was analyzed at the High Performance Liquid Chromatography (HPLC) laboratory; Medical Research Center in the Faculty of Medicine, Alexandria University, Waters Alliance e2695 apparatus was used. An HPLC complete kit from Recipe^®^ together with a C18 analytical column and a UV detector was used (Barshop and Gangoiti, 2007). The recoveries and concentrations of the calibrator, low control and high control were calculated and compared with the standard references provided- System suitability check (Figure 1). Once the results were found acceptable, the samples were prepared and run. The chromatograms were integrated and the concentrations were calculated (Table 1).

*Spectrophotometric assay*

TAC was assayed spectrophotometrically using a Biodiagnostics^®^ kit (Catalogue number TA 25 13). The determination is performed by the reaction of the antioxidants in the sample with a known excess of exogenously provided hydrogen peroxide (H_2_O_2_) (Koracevic etal, 2001). The TAC eliminates a certain amount of H_2_O_2_ equivalent to its concentration, leaving a residual amount of H_2_O_2_ that is determined colorimetrically, by an enzymatic reaction involving conversion of 3,5, dichloro–2–hydroxy benzensulphonate to a colored product. 

ICP-OES: ICP-OES 5100 VDV, Agilent Technologies, was used for the determination of trace elements, Cu and Zn, in the serum samples by inductively coupled plasma-optical emission spectrometry. 48 breast cancer patients and 16 control subjects were analyzed. The instrumental operating conditions are enlisted in Table 2. Sample preparation, elements analysis and quality control were carried out according to standard methods US EPA Method 200.7 and US EPA Method 6010 C. Results were expressed in ppm (µg/ml).

*Statistical analysis*

Mann Whitney test was used to evaluate non parametric data and Student t-test was used for parametric data analysis using a 95% confidence interval. Being a case-control study, we used the odds ratio to measure the effect or the impact of exceeding specified limits of TAC and CoQ10 on the risk of developing breast cancer. Stepwise multiple regression analysis was conducted to test the variables contributing in the serum TAC and CoQ10 levels. The independence of variables was assessed using dubin-watsin test. Multi-colinearity was assessed using Variance influence factor (VIF), Tolerance and Correlation matrices. Presence of outliers and influencers were also assessed (Kotz et al., 2006; Kirkpatrick and Feeney, 2013). 

## Results

The control group had a median age of 38.50 (32.0- 52.0) years and the breast cancer group had a median age of 41.50 (26.0 – 55.0) years. The BMI in the control group was significantly lower than the breast cancer group 25.75 (21.26 – 37.80) kg/m^2^ and 29.34 (23.18 – 39.78) kg/m^2^ respectively. Most of the breast cancer patients (93.3%) were of early stages (II and IIIa). Tumor size T2 with a nodular invasion of N1 were seen in 61.7% of them. None of the breast cancer patients showed evidence of metastasis. 

No statistically significant difference was found among the studied groups regarding the serum levels of random glucose, creatinine and aminotransferase activities while there was a statistically significant increase in the urea levels and a decrease in serum ALP activity in the control group when compared to the breast cancer patients, however, both lie within the reference values for age and sex. The CA 15-3 was significantly higher in the breast cancer group 23.90 (11.0-300.0) U/mL when compared to the control group 7.0 (4.50-16.0) U/mL. There was a statistically significant increase in the CoQ10 and TAC levels in the breast cancer group when compared to the control group (Table 3).

Using the odds ratio, it was found that subjects with TAC values >1.5 mMol/L were 2.2 times more at risk of developing breast cancer than those with TAC values ≤1.5 mMol/L with a p value of 0.049 at a 95% confidence interval. It was also found that subjects with CoQ10 >1600 µg/L were 7.9 times more at risk of developing breast cancer than those with CoQ10 values <1600 µg/L with a borderline significance p of 0.053 at a 95% confidence interval (Table 4).

Multiple linear regression analysis performed nullified the effect of the BMI and has found TAC to be an independent predictor of breast cancer. The adjusted odds ratio also found TAC to be statistically significant upon nullifying the effect of BMI. It was found to be 5.944 (1.203-29.371) with a p value of 0.029 at a 95% confidence interval. 

The levels of copper were significantly higher in the breast cancer group (1.18 (0.55-2.79) µg/ml) when compared to the control group (0.81 (0.50-1.98) µg/ml). The levels of zinc showed no significant difference between the breast cancer group (1.64 (0.49-4.79) µg/ml) and the control group (1.29 (0.26-3.18) µg/ml).

**Table 1:** Low Control, High Control Chromatogram and System Suitability Check

Sample	Area of IS	Recovery	Concentration of CoQ10 (μg/L)	Accepted range (μg/L)
Calibrator	18,619		918	
Low control	16,762	0.900	439.81	414- 620
High control	16,892	0.907	1288.55	1,058-1,586

**Table 2:** The ICP-OES Instrumental Operating Conditions for the Determination of Trace Elements

RF power (kW)	1.2
Viewing mode	Axial
Sample introduction	Manual
Sample Uptake Time (s)	10
Stabilization time (s)	10
Nebulizer flow (L/min)	0.7
Plasma flow (L/min)	12
Aux flow (L/min)	1

**Table 3:** CoQ10 and TAC Levels in the Breast Cancer Group Compared to the Control Group

Parameters	Test (P)
CoQ10 (µg/L) N	U=372*(0.046*)
TAC (mMol/L)	t=2.1 (0.044)*

U, Mann-Whitney test statistics for non-parametric data analysis; t, student t-test statistics for parametric data analysis; *, p-values ≤ 0.05 were considered statistically significant

**Figure 2.**
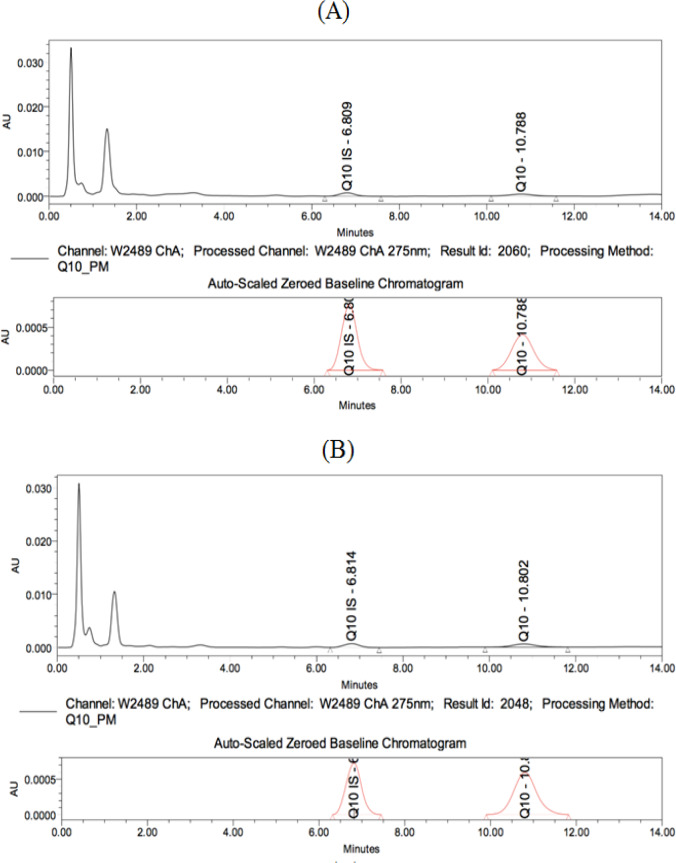
Coenzyme Q10 Chromatograms of a Control Serum Sample (A) versus a breast cancer serum sample (B)

**Table 4:** Risk of Developing Breast Cancer Based on Levels of Q10 and TAC

	Controls		Patients		OR	95% C.I
	No.	%	No.	%		
TAC	(n= 20)		(n= 60)			
≤1.5 mMol/L	1	5	18	30	-	-
>1.5 mMol/L	19	95	42	70	2.211*	1.012 – 65.526
Q10	(n= 18)		(n= 60)			
<1,600 µg/L	17	94.4	41	68.3	-	-
>1,600 µg/L	1	5.6	19	31.7	7.878	0.976 –63.260

OR, Odds Ratio; C.I, Confidence interval; b, Null hypothesis; true area, 0.5

## Discussion

In the present study a statistically significant increase was found in the TAC levels in the breast cancer patients (1.80 ± 0.35 mMol/L) when compared to the control group (1.62 ± 0.29 mMol/L). Females having TAC levels above 1.5 mMol/L were 2.2 times more at risk of developing breast cancer than those with values below 1.5 mMol/L with odds ratio 2.211 (1.012-65.526) with a 95% confidence interval and p value of 0.049. The multiple linear regression analysis performed nullified the effect of the BMI and found TAC to be an independent predictor of breast cancer. The adjusted odds ratio has also found TAC to be statistically significant upon nullifying the effect of BMI. It was found to be 5.944 (1.203-29.371) with a p value of 0.029 at a 95% confidence interval.

In agreement with our results, Gonenc et al., (2006) found a raised antioxidant status in breast cancer tissue when compared to benign breast diseased tissue in a study performed on 15 females newly diagnosed breast cancer patients and 15 female patients with benign breast disease. A study conducted by Portakal et al., (2000) carried out on 21 breast cancer women found a significantly increased expression of superoxide dimustase (SOD), catalase (CAT) and glutathione peroxidase (GPx) in breast cancer tissue of patients in comparison to the cancer free tissue levels in the same patients. Another study evaluated the status of free radical production and different antioxidants on 54 non-intervened breast cancer patients and found a significantly increased free radical production, GPx and SOD activity and a reduced CAT activity in breast cancer patients when compared to the control group (Ray et al., 2000). Rajneeshet al., (2008) carried out the study on 40 newly diagnosed breast cancer patients and 100 healthy control subjects found significantly increased levels of glutathione (GSH), SOD, CAT, GPx concentrations and GST activities in breast cancer patients when compared to the control group. Moreover, a study performed on 136 breast cancer patients and 183 control subjects taking the study in the years 2007–2012 showed significantly higher GPx activity and thiobarbituric acid reactive substances (TBARS; the byproduct of lipid peroxidation) in the breast cancer patients when compared to the healthy controls. 

On the other hand, a case control study performed by Ching et al., (2002) on 153 breast cancer patients and 151 healthy controls, evaluating total antioxidant status as a predictor of breast cancer, has found an increased breast cancer risk with a decreased serum level of total antioxidant capacity. Sener et al., (2007) carried out his study on 56 breast cancer patients and 18 healthy women and has found a significantly lower TAC in breast cancer patients. Badid et al., (2010) also found a decreased total antioxidant status in breast cancer patients in 38 newly diagnosed breast cancer patients and 50 healthy controls. Another study performed by Gupta et al., (2012) on 30 females recently diagnosed breast cancer patients and 100 healthy females as controls found a significantly lower SOD and GPx activity and lower serum TAC levels in breast cancer patients when compared to the control group. This decrease in antioxidants was explained to be as a consequence of the enhanced accumulation of free radicals. 

A study conducted by Omar et al., (2011) on 40 breast cancer patients taking chemotherapy with a control group of 20 subjects and another study conducted by Panis et al., (2012) on 60 breast cancer patients with advanced ductal infiltrative carcinoma treated with doxorubicin or paclitaxel and a control group of 30 healthy controls have found similar findings. Omar ME et al found a significantly lower total antioxidant capacity in breast cancer patients versus the healthy controls and Panis C et al has found a significantly lower CAT, SOD and GSH activity in the breast cancer patients when compared to the control group. The present study nullifies the effects attributed to chemotherapeutic agents or surgery, as all our patients were non-intervened. 

Several possible explanations can justify an increased level of TAC in the breast cancer group when compared to the control group. Redox adaptation through the elevation of endogenous antioxidants and the activation of cell survival pathways may confer an increased capacity to tolerate exogenous stress and insults, a decrease in apoptotic execution and elevated DNA repair capability (Trachootham et al., 2009). Higher antioxidant enzymes were also thought to lead to an increase in the cure rate of breast cancer (Portakal et al., 2000). This finding might be the reason behind a better prognosis to the breast cancer patients in the present study that indeed showed no signs of metastasis. Moreover, earlier reports have also found an increase in antioxidant enzymes’ activities in tumor tissues dependent upon overexpression of the enzymes (Liu et al., 1997). Hence the increase in TAC in the present study may also be due to an increase in enzyme expression in tumor cells. 

In the present study the CoQ10 levels were significantly higher in the breast cancer group (1337.67 (630.36-3333.51) µg/L) when compared to the control group (1195.30 (647.58–1775.01) µg/L). A CoQ10 level above 1600 µg/L was found to have an odds ratio of 7.878 (0.976-63.260) with a 95% confidence interval which possessed a risk for breast cancer development but the significance was compromised (p= 0.053). Yet, a larger sample size could clarify these findings further. In accordance to the results of the present study a large prospective study performed by Chai et al., (2010) found a positive association for circulating levels of total CoQ10 and breast cancer risk. This study proposed a possible role for CoQ10 in the development and progression of breast cancer but epidemiologic evidence is lacking. Circulating CoQ10 may not be indicative of intracellular CoQ10 yet may be a response to chronic inflammation, heightened systemic or tissue-specific oxidation (Menke et al., 2008). On the other hand, Jolliet et al., (1998) found decreased levels of CoQ10 in breast cancer patients in both malignant and benign tumors. Portakal et al., (2000) has also showed significantly reduced CoQ10 levels in breast cancer tissue when compared to healthy tissue of the control group. Another prospective study perfomed by Cooney et al., (2011) on CoQ10 in breast cancer patients found opposing results to the prospective study mentioned earlier. A borderline significant inverse relationship between CoQ10 levels and breast cancer incidence was found. 

Several reasons can be used to explain the increased CoQ10 levels in breast cancer patients when compared to the healthy controls. Initially, low circulating levels of CoQ10 have been associated with poor prognosis for a number of cancer types. Also, patients who developed metastasis had lower CoQ10 levels than those who did not and subjects with lower baseline CoQ10 levels had shorter disease free intervals (Rusciani et al., 2006). These findings might link the high levels of CoQ10 found in the present study to the fact that most of the breast cancer patients were in the early stage of the disease and none of them showed signs of metastasis which is consistent with the fact that low circulating levels of CoQ10 were associated with poor prognosis. It’s also noteworthy that women at either extreme values of CoQ10 may be at increased risk of breast cancer (Cooney et al., 2011). This has been demonstrated in the present study where CoQ10 levels lied at the higher extreme values and breast cancer patients with CoQ10 values >1600 µg/L showed 7.9 times the odds of being at risk of developing breast cancer. Moreover, the lower levels of CoQ10 found in the prospective study performed by Cooney et al., (2011), might not contradict the findings of the prospective study performed by Chai et al., (2010), yet may be suggestive of a possible nonlinear (U-shaped) association of CoQ10 with breast cancer risk. These findings add to the controversial debate about the levels of CoQ10 in breast cancer.

In the present work, the levels of copper were significantly higher in the breast cancer group (1.18 (0.55-2.79) µg/ml) when compared to the control group (0.81 (0.50-1.98) µg/ml). The levels of zinc showed no significant difference between the breast cancer group (1.64 (0.49-4.79) µg/ml) and the control group (1.29 (0.26-3.18) µg/ml). (Pavithra et al., 2015), has found a statistically significant increase in copper levels in breast cancer group when compared to control group, yet a decrease in zinc levels in breast cancer group when compared to the control group. (Feng et al., 2011) has found a significantly increased level of copper in breast cancer group (1159.3 + 144.7) µg/L when compared to the control group (1016.2 + 100.2 ) yet statistically significant decrease in zinc in breast cancer group ( 939.1+ 103.3) µg/L when compared to the control group (1060.9 +86.8) µg/L. (Saleh et al., 2010), has found a significantly lower level of copper (1.33 ± 0.34 in breast cancer patients vs 1.47 ± 0.45 in controls) and zinc in breast cancer patients (0.99 ± 0.39mg/L) when compared to healthy controls 3.6 ± 1.1 mg/L. 

In conclusion, we highlight the increase in TAC, CoQ10 and copper levels in the breast cancer group when compared to the control group. It is still unclear whether the increased antioxidant defenses offer a selective growth advantage to tumor cells over their surrounding normal cells or serve as a protective measure by the body in an attempt to correct the assault triggered by the ROS. The conflicting findings from the studies has left a gap for more extensive research to evaluate the effect of the disease course on the antioxidants’ levels and the effect of the antioxidants’ levels on the onset and progression of breast cancer.
